# Breast cancer is associated with coronary heart disease: a cross-sectional survey of NHANES 1999–2018

**DOI:** 10.3389/fcvm.2023.1274976

**Published:** 2023-12-06

**Authors:** Luyao Dai, Ruoxuan Li, Qian Hao, Yuanhang Bao, Liqun Hu, Yaohui Zhang, Huafeng Kang, Hao Wu, Xiaobin Ma, Yafan Song

**Affiliations:** ^1^ Department of Oncology, The Second Affiliated Hospital of Xi’an Jiaotong University, Xi’an, Shaanxi, China; ^2^ College of Art & Science, Boston University, Boston, MA, United States; ^3^ School of Basic Medical Sciences, Xi’an Key Laboratory of Immune Related Diseases, Xi’an Jiaotong University, Xi’an, Shaanxi, China; ^4^ Department of Cardiology, The Second Affiliated Hospital of Xi’an Jiaotong University, Xi’an, Shaanxi, China

**Keywords:** the National Health and Nutrition Examination Survey, breast cancer, coronary heart disease, risk factors, prevention

## Abstract

**Background:**

Understanding the correlation between female breast cancer (BC) and the prevalence of coronary heart disease (CHD) is important for developing prevention strategies and reducing the burden of female social disease. This study aimed to evaluate the relationship between BC and CHD using data from the National Health and Nutrition Examination Survey (NHANES) database from 1999 to 2018.

**Methods:**

The study cohort included 16,149 eligible non-pregnant female participants aged 20 years or older. Logistic regression was used to analyze the relationship between BC and CHD, excluding the interaction between covariates and BC through hierarchical subgroup analysis.

**Results:**

The study found that participants with BC had a 2.30 times greater risk of developing CHD compared to those without BC [95% confidence interval (CI): 2.29–2.31]. After adjusting for all included covariates, BC was still significantly associated with CHD risk (odds ratio: 1.11, 95% CI: 1.10–1.12). When participants were stratified by age, education level, and prevalence of hypertension, it was evident that participants with BC had a higher risk of developing CHD compared to those without BC, although the effect of BC on CHD varied across stratification.

**Conclusions:**

Our study demonstrates the close relationship between CHD and female BC. Therefore, it is necessary to screen patients with CHD for BC and monitor BC survivors for the long-term risk of developing CHD.

## 1. Introduction

Cardiovascular disease (CVD) is a leading cause of death worldwide, accounting for approximately one-third of annual deaths (1). In recent years, the risk and incidence rate of CVD have been on the rise due to factors such as people's insufficient physical activity and air pollution (2). Among CVD cases, coronary heart disease (CHD) is the leading cause of death (3, 4). Traditional risk factors for CHD include age, hypertension, diabetes, smoking, dyslipidemia, obesity, and a family history of early CHD. However, these traditional risk factors alone are no longer sufficient to explain the entire risk of CHD events. Given the high incidence and health burden of CHD, there is an urgent need to explore new risk factors to prevent its occurrence and development (5, 6).

Cancer is another major cause of death besides CVD. The latest global cancer burden shows that cancer incidence is increasing worldwide (7). Nevertheless, cancer screening, diagnosis, and treatment advances have reduced cancer-specific mortality (8). In this context, as cancer survivors live longer, their risk of death from other causes increases. Numerous studies have shown that cancer survivors are more likely to develop and die from CVD than the general population (9–11).

Recent evidence suggests that cancer and CHD share common risk factors and mechanistic links. Overlapping risk factors for the two diseases include age, obesity, smoking, and diabetes. Additionally, regarding pathophysiological mechanisms, inflammation can promote the occurrence and development of cancer and CHD by mediating the production of ROS. Nevertheless, further studies are needed to evaluate the role of anti-inflammatory therapy in cancer and CHD (12).

In 2020, female breast cancer (BC) surpassed lung cancer as the most common cancer worldwide. BC is also the leading cause of cancer-related death in women (7). Over the past few decades, improvements in screening and treatment techniques for women with BC have increased breast cancer-specific survival rates. Nevertheless, on the other hand, non-cancer mortality (such as CHD) also increased in BC patients (13). Understanding the correlation between BC and CHD prevalence and formulating corresponding prevention strategies are essential to reduce the social disease burden of women. Few sizeable population-based cohort studies have been explicitly used to explore the relationship between BC and CHD. The National Health and Nutrition Examination Survey (NHANES) is one of the most representative large-scale surveys based on the United States (US) population. This study was designed to assess the relationship between BC and CHD using data from the NHANES from 1999 to 2018.

## 2. Methods

### 2.1. Study population

NHANES is a population-based cross-sectional survey designed to collect information on the health and nutritional status of the US population. Researchers used a complex stratified multi-stage sampling design to obtain a representative sample of the US population. The NHANES study was approved by the National Center for Health Statistics (NCHS) Research Ethics Review Board. Each participant signed a written informed consent form (14).

**Figure 1:**
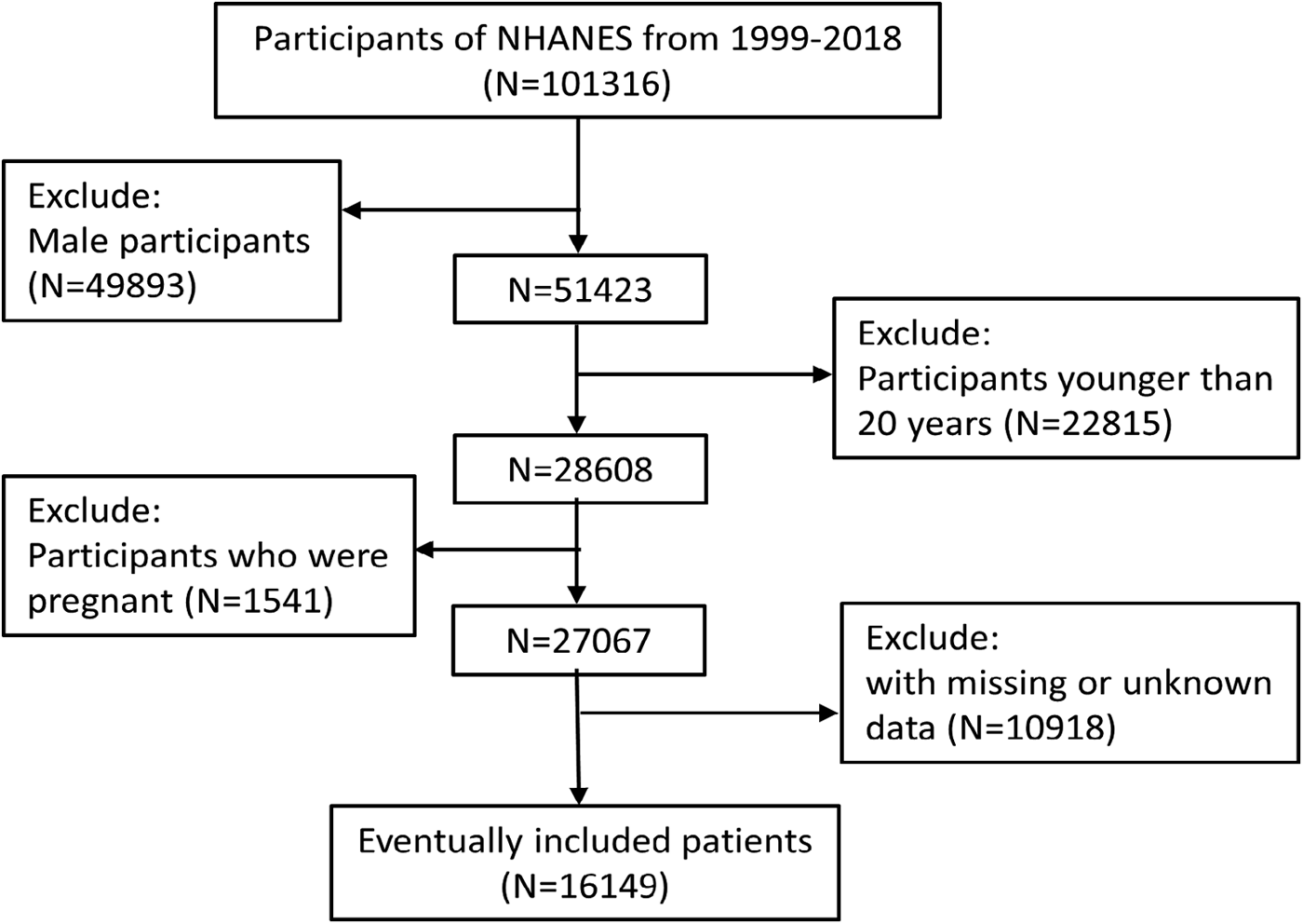
Flow chart yof the sample selection from the NHANES 1999–2018.

All data used in this study were from the NHANES database. We downloaded 20 years of public data from the NHANES website from 1999 to 2018. A total of 101,316 participants completed the survey over the ten survey periods. After excluding males (49,893), individuals under 20 years of age (22,815), pregnant individuals (1,541), and individuals with missing or unknown information (10,918), 16,149 subjects were ultimately enrolled for analysis. The detailed screening process for participants included in this study was shown in Figure 1.

### 2.2. Diagnosis of breast cancer

The self-reported diagnosis of BC came from the medical condition questionnaires. Participants were asked: “Have you ever been told by a doctor or other health professional that you had cancer or a malignancy of any kind?”. Participants who answered “yes” were then asked, “What kind of cancer was it?”. Respondents who reported only BC (primary and single tumor) were classified as BC patients. Any respondent who answered “no” or reported a history of other cancers or reported a history of BC combined with other cancers was considered a non-BC population.

### 2.3. Diagnosis of coronary heart disease

Similarly, the diagnosis of CHD also came from the medical history review of the medical condition questionnaires. Participants were asked: “Has a doctor or other health professional ever told you that you had coronary heart disease?” Those who answered “yes” to this question were considered to have CHD, and the rest were considered to have no CHD.

### 2.4. Covariates

Participants were divided by age into two groups: <50 years old and ≥50 years old. Marital status was divided into married and unmarried (including single, separated, divorced, widowed, or domestic partner) groups. Race was divided into non-Hispanic white, non-Hispanic black, Mexican American, and other. Education level was divided into below high school, high school level or equivalent, and above high school. The poverty-to-income ratio (PIR) is calculated by dividing household income by the poverty threshold for the survey year (15). We divided PIR into ≤1, 1–3, and ≥3. Hypertension was recorded as a diagnosis history informed by a doctor or other health professional or being on prescription medication for hypertension. A diagnosis of hypercholesterolemia informed by a doctor or other health professional was defined as having hypercholesterolemia. The diagnosis of diabetes came from a self-reported doctor's diagnosis or being treated with medication or insulin. People who smoked less than 100 cigarettes in their lifetime or who did not smoke were grouped and defined as non-smokers. Smokers were current smokers or those who had smoked more than 100 cigarettes. Non-drinkers drank no more than 12 glasses of alcohol a year, and those who drank more were considered drinkers. Physical activity was divided into self-reported vigorous/moderate physical and non-physical recreation. Body mass index (BMI) is calculated by dividing weight (kg) by the square of height (m^2^) (16). Finally, the daily intake data of energy, carbohydrate, protein, total fat, fiber, folate, vitamin B12, sodium, and caffeine were obtained through 24-hour dietary recall.

### 2.5. Statistical analysis

Given NHANES’ complex survey design characteristics, all data were weighted according to the NHANES analysis guidelines (14). Weighted mean ± standard error or weighted median (interquartile spacing) was used to represent continuous data, and unweighted numbers (weighted percentage, %) were used to represent classified data. The logistic regression model was used to study the relationship between BC and CHD, and odds ratio (OR) and 95% confidence interval (95% CI) were used to quantify the relationship. We tested the regression model by gradually adjusting for potential confounding factors. Model 1 was unadjusted. Model 2 was adjusted for age, marital status, race/ethnicity, education level, and PIR. Model 3 was further adjusted for smoking, alcohol intake, physical activity, and BMI. Model 4 further adjusted hypertension, hypercholesterolemia, and diabetes based on Model 3. Model 5 adjusted dietary factors, including energy, carbohydrate, protein, total fat, fiber, folate, vitamin B12, sodium, and caffeine, based on Model 4. Additionally, subgroup analysis was performed according to age, education level, and hypertension, and all covariates were adjusted according to Model 5 (except grouping covariates). All statistical analyses were performed using SPSS (Version 25). Bilateral *p* < 0.05 was considered statistically significant.

## 3. Results

### 3.1. Baseline characteristics of respondents

Table 1 showed the baseline characteristics of all participants (16,149 in total) in this study. Compared with people without BC, those with BC were more likely to be ≥50 years old, married, non-Hispanic white, PIR ≥3, and have hypertension. For those without BC, more people had more education than high school, did not have hyperlipidemia and diabetes, did not smoke but drank alcohol, and engaged in physical activity than those with BC (*P* < 0.001 for all). In addition, BMI and daily intakes of energy, carbohydrates, protein, total fat, and sodium were lower in patients with BC than in patients without BC. In contrast, BC patients’ daily dietary fiber, folic acid, vitamin B12, and caffeine intake were higher than those without BC.

**Table 1:** Baseline characteristics of the study participants by different diseases (*N* = 16,149)^a^.

Variable	Breast cancer			Coronary heart disease		
	Yes (*N* = 516)	No (*N* = 15,633)	*P*	Yes (*N* = 472)	No (*N* = 15,677)	*P*
Age (year)			<0.001			<0.001
< 50	35 (6.8)	7,284 (46.6)		30 (6.4)	7,289 (46.5)	
≥50	481 (93.2)	8,349 (53.4)		442 (93.6)	8,388 (53.5)	
Marital status			<0.001			<0.001
Married	255 (56.9)	7,609 (54.9)		187 (44.4)	7,677 (55.2)	
Unmarried	261 (43.1)	8,024 (45.1)		285 (55.6)	8,000 (44.8)	
Race/ethnicity			<0.001			<0.001
Non-Hispanic white	328 (85.5)	7,350 (71.6)		302 (80.0)	7,376 (71.8)	
Non-Hispanic black	86 (6.9)	3,353 (10.8)		70 (7.1)	3,369 (10.7)	
Mexican American	45 (2.8)	2,258 (6.3)		44 (2.7)	2,259 (6.3)	
Other	57 (4.8)	2,672 (11.3)		56 (10.3)	2,673 (11.1)	
Education level			<0.001			<0.001
Below high school	102 (12.3)	3,366 (13.6)		164 (27.1)	3,304 (13.2)	
High school or equivalent	118 (24.2)	3,476 (22.8)		137 (31.0)	3,457 (22.6)	
Above high school	296 (63.5)	8,791 (63.6)		171 (41.9)	8,916 (64.2)	
Poverty to income ratio			<0.001			<0.001
≤1	76 (8.3)	2,990 (13.4)		114 (16.7)	2,952 (13.1)	
1–3	212 (35.4)	6,516 (35.3)		239 (49.3)	6,489 (35.0)	
≥3	228 (56.3)	6,127 (51.3)		119 (34.0)	6,236 (51.9)	
Hypertension			<0.001			<0.001
Yes	302 (51.3)	6,155 (34.1)		381 (80.7)	6,076 (33.4)	
No	214 (48.7)	9,478 (65.9)		91 (19.3)	9,601 (66.6)	
Hypercholesterolemia			<0.001			<0.001
Yes	268 (48.6)	5,954 (36.1)		361 (77.2)	5,861 (35.5)	
No	248 (51.4)	9,679 (63.9)		111 (22.8)	9,816 (64.5)	
Diabetes			<0.001			<0.001
Yes	106 (15.8)	2,100 (9.7)		178 (38.5)	2,028 (9.2)	
No	410 (84.2)	13,533 (90.3)		294 (61.5)	13,649 (90.8)	
Smoking			<0.001			<0.001
Yes	220 (43.0)	5,878 (40.1)		249 (55.4)	5,849 (39.8)	
No	296 (57.0)	9,755 (59.9)		223 (44.6)	9,828 (60.2)	
Alcohol intake			<0.001			<0.001
Yes	284 (62.3)	9,052 (65.5)		216 (49.8)	9,120 (65.8)	
No	232 (37.7)	6,581 (34.5)		256 (50.2)	6,557 (34.2)	
Physical activity			<0.001			<0.001
Yes	237 (52.8)	7,926 (57.5)		165 (43.9)	7,998 (57.7)	
No	279 (47.2)	7,707 (42.5)		307 (56.1)	7,679 (42.3)	
BMI (kg/m^2^)	28.8 ± 6.7	29.2 ± 7.6	<0.001	30.0 ± 6.9	29.2 ± 7.5	<0.001
Energy (kcal)	1,745.8 ± 630.2	1,903.2 ± 725.4	<0.001	1,577.3 ± 695.8	1,807.2 ± 722.4	<0.001
Carbohydrate (g)	211.8 ± 91.3	218.9 ± 97.8	<0.001	197.1 ± 84.9	219.3 ± 97.9	<0.001
Protein (g)	61.1 (47.0,82.3)	64.6 (47.3,85.3)	<0.001	55.7 (48.8,70.9)	64.7 (47.6,85.6)	<0.001
Total fat (g)	61.7 (46.3,85.5)	64.4 (44.7,89.3)	<0.001	56.0 (38.4,75.5)	64.7 (45.0,89.5)	<0.001
Fiber (g)	13.9 (9.9,20.0)	13.4 (8.9,19.2)	<0.001	11.8 (8.4,17.5)	13.5 (9.0,19.3)	<0.001
Folate (µg)	315.0 (214.0,413.0)	308.0 (215.0,444.0)	<0.001	278.0 (186.0,402.0)	310.0 (215.0,444.0)	<0.001
Vitamin B12 (µg)	3.4 (1.9,5.6)	3.3 (1.9,5.3)	<0.001	2.8 (1.7,4.4)	3.3 (1.9,5.3)	<0.001
Sodium (g)	2.7 (2.0,3.4)	2.8 (2.0,3.7)	<0.001	2.4 (1.7,3.2)	2.8 (2.0,3.7)	<0.001
Caffeine (mg)	116.0 (27.0,239.0)	115.0 (29.0,231.0)	<0.001	105.0 (11.0,225.0)	115.0 (30.0,232.0)	<0.001

Unmarried: single (never married), separated, divorced, widowed, or domestic partner; BMI, body mass index.

^a^ Continuous variables were represented by weighted mean ± standard error or weighted median (interquartile range), and categorical variables were represented by unweighted numbers (weighted percentage, %).

Compared with people without CHD, those with CHD were more likely to be ≥50 years of age, unmarried, non-Hispanic white, with less than a high school education, PIR ≤1, and have diabetes (all *P* < 0.001). Most CHD patients smoked but did not drink alcohol or engage in physical activity, and they also suffered from hypertension and hyperlipidemia. In contrast, most people who did not have CHD did not smoke or have hypertension or hyperlipidemia but did drink alcohol and did physical activity. Additionally, patients with CHD had a higher BMI than those without CHD (*P* < 0.001). Regarding diet, the average daily intake of energy, carbohydrate, protein, total fat, fiber, folate, vitamin B12, sodium, and caffeine in CHD patients was lower than those without CHD, and the differences were statistically significant.

### 3.2. Association of BC with CHD

Table 2 detailed the relationship between BC and CHD. Among the five logistic regression models established, the high risk of CHD was significantly correlated with BC (all *P* < 0.001). Unadjusted Model 1 showed that patients with BC had a 2.30-fold increased risk of CHD compared with those without BC (OR: 2.30, 95% CI: 2.29–2.31). We gradually controlled covariables from Model 2 to Model 5 to reduce potential confounding factors. As more covariates were controlled, the correlation between BC and CHD prevalence gradually decreased. However, it was worth mentioning that although the 95%CI of Model 5 was different from that of Model 4, their OR values were the same, indicating that dietary factors controlled by Model 5 had little influence on the relationship between BC and CHD. Model 5 completely adjusted for all covariates in this study, including age, marital status, race/ethnicity, education level, PIR, smoking, alcohol intake, physical activity, BMI, hypertension, hypercholesterolemia, diabetes, energy, carbohydrate, protein, total fat, fiber, folate, vitamin B12, sodium, and caffeine. Finally, BC was still significantly associated with increased CHD prevalence (OR: 1.11, 95% CI: 1.10–1.12).

**Table 2:** Association between breast cancer and coronary heart disease.

	Model 1 OR (95% CI)	Model 2 OR (95% CI)	Model 3 OR (95% CI)	Model 4 OR (95% CI)	Model 5 OR (95% CI)
Non-breast cancer	Reference	Reference	Reference	Reference	Reference
Breast cancer	2.30 (2.29–2.31)	1.17 (1.17–1.18)	1.18 (1.18–1.19)	1.11 (1.10–1.11)	1.11 (1.10–1.12)

Model 1: Non-adjusted.

Model 2: Adjusted for age, marital status, race/ethnicity, education level, and poverty to income ratio.

Model 3: Further adjusted for smoking, alcohol intake, physical activity, and BMI.

Model 4: Further adjusted for chronic disease including hypertension, hypercholesterolemia, and diabetes.

Model 5: Further adjusted for dietary factors including energy, carbohydrate, protein, total fat, fiber, folate, vitamin B12, sodium, and caffeine.

### 3.3. Subgroup analysis

Based on the results in Tables 1, 2, and clinical experience, subgroup analysis was stratified by age, education level, and the presence or absence of hypertension. All covariables were adjusted according to Model 5 (those used for grouping were not adjusted). The analysis results in Table 3 showed that regardless of which covariate was used for stratification, BC and CHD had a significant correlation (all *P* < 0.001). In the <50 age group, the risk of CHD in BC patients (OR: 2.11, 95% CI: 2.01–2.22) was higher than that in the ≥50 age group (OR: 1.24, 95% CI: 1.23–1.24). After stratification by education level, the higher the degree of education, the higher the risk of CHD in BC patients. Interestingly, the risk of CHD in BC patients with hypertension (OR: 1.15, 95% CI: 1.15–1.16) was lower than in BC patients without hypertension (OR: 1.56, 95% CI: 1.54–1.58).

**Table 3:** Subgroup analysis of association between breast cancer and coronary heart disease.

Subgroups	Non-breast cancer OR	Breast cancer OR (95% CI)	*P*
Age (year)			
<50	Reference	2.11 (2.01–2.22)	<0.001
≥50	Reference	1.24 (1.23–1.24)	<0.001
Education level			
Below high school	Reference	1.13 (1.11–1.15)	<0.001
High school or equivalent	Reference	1.16 (1.15–1.17)	<0.001
Above high school	Reference	1.32 (1.31–1.33)	<0.001
Hypertension			
Yes	Reference	1.15 (1.15–1.16)	<0.001
No	Reference	1.56 (1.54–1.58)	<0.001

All data were adjusted for age, marital status, race/ethnicity, education level, poverty to income ratio, smoking, alcohol intake, physical activity, BMI, hypertension, hypercholesterolemia, energy, carbohydrate, protein, total fat, fiber, folate, vitamin B12, sodium, and caffeine (except for grouping covariates).

## 4. Discussion

CVD and cancer are the two most common causes of morbidity and mortality in developed countries. The growing global burden of cancer and CVD has become a significant public health concern (17). The most effective strategy for primary prevention or management of CHD will likely be achieved by modifying its risk factors. Identifying and avoiding risk factors for CHD is essential to prevent and reduce the development of CHD. In addition, multidisciplinary research is needed to improve the understanding of CHD, including the causes, triggers, pathogenesis, and effective preventive management measures.

Previous studies have shown that cancer survivors have an increased risk of CVD. Although cardiovascular care management for BC survivors is evolving, there is a knowledge gap in the epidemiological results of CHD in these patients (18). Our cross-sectional study included data from 16,149 participants who participated in NHANES from 1999 to 2018. The results showed a positive association between BC and CHD in a nationally representative population of the US. This association was independent of other confounding factors, including age, marital status, race/ethnicity, education level, PIR, smoking, alcohol intake, physical activity, BMI, hypertension, hypercholesterolemia, diabetes, energy, carbohydrate, protein, total fat, fiber, folate, vitamin B12, sodium, and caffeine. In the fully adjusted model, participants with BC had a higher risk of developing CHD, with OR and 95% CI of 1.11(1.10–1.12). Subsequent subgroup analyses also showed that the OR associated with BC and CHD was >1, even after stratification using covariates such as age, education, and hypertension. Our results provided evidence for an association between BC and CHD.

The age-stratified analysis showed a significant correlation between BC and CHD. However, the association between BC and CHD did not increase with age. In contrast, the extent of the association decreased among older participants. According to literature reports, the correlation between most risk factors and biomarkers of CHD and CHD weakens with increasing age of onset (19), which was consistent with our findings. Additionally, the American Heart Association survey showed that younger women were more likely to identify multiple barriers to a heart-healthy lifestyle, including adverse psychological events such as stress (20). Stress is also a risk factor for BC (21). This made sense as to why young BC survivors were more likely to develop CHD.

In the stratified analysis of educational attainment, there was still a significant correlation between BC and CHD. The correlation degree gradually increased from 1.13 to 1.32 with increased educational background. A Mendelian randomization study in 2017 showed that more extended education could reduce the risk of CHD (22). Similarly, the results of a 2019 analysis also supported that education could prevent the risk of CHD in the general population (23). However, in our study, the more education BC patients had, the more likely they were to develop CHD. Several studies have shown that the incidence of BC was positively correlated with the level of education (24, 25). In our baseline table, most BC patients (63.5%) had a high school education or above. One reason for this positive association may be due to more frequent screening among people with more education (26). In other words, the chances of these people being screened for CHD will also increase. On the other hand, BC patients with higher education have better cancer survival compared to BC patients with less education (27). These populations also have an increased risk of developing other diseases (including CHD) during the more extended survival period.

The subgroup analysis results also showed that BC survivors without hypertension had a higher risk of developing CHD compared to BC survivors with hypertension. This seemed to be contrary to people's expectations, as hypertension was often considered beneficial for the development of CHD (28). In our study cohort, hypertension in the enrolled population may have been preexisting or newly developed in the context of cancer treatment. In fact, hypertension is one of the most common complications among cancer patients. Many anti-tumor drugs or therapies have antihypertensive effects (29). Therefore, BC patients with hypertension may have actively undergone anti-tumor treatment. Moreover, the potential cardiovascular toxicity of traditional chemotherapy drugs (such as Anthracycline and antimetabolic drugs) is prominent. That is to say, hypertension may expose cancer survivors to the risk of cardiovascular adverse events. Although this contradicts the results of our analysis, studies have shown that low blood pressure (<120 or <70 mmHg) is also associated with increased cardiovascular outcomes and death (30). Furthermore, due to the nature of the retrospective analysis applied in this study, these results should be interpreted cautiously. Because of increasing awareness of the association between hypertension levels after anti-cancer treatment and an increased risk of cardiovascular outcomes, cardiovascular health management for survivors is also being optimized. Further preclinical and clinical studies are needed to explore the effect of hypertension on CHD in BC survivors.

Although much published literature has reported the connection between cancer and CHD in the past few decades, the underlying mechanisms seem complex and multidimensional (31, 32). Firstly, chronic inflammation is a recognized common feature of the pathogenesis of CHD and cancer (33, 34). Therefore, the risk factors inducing inflammation (including obesity, dyslipidemia, hypertension, and diabetes) are also common risk factors for CHD and cancer (35). Given this, some scholars have used the commonly used 10-year risk score for CHD to predict the risk of future cancer occurrence (36). Secondly, some evidence suggests that metabolism is a central mechanism in CHD and cancer. In the cardiovascular system, metabolic changes are associated with dysfunction or maintenance of tissues and organs, while in the cancer system, metabolic remodeling supports the occurrence and development of malignancies. In addition, shared molecular and genetic pathways (such as adenosine 5’ monophosphate-activated protein kinase, LRP6 mutation, and Wnt signaling pathway) may also explain the similarities between CHD and cancer (37).

The multi-center and prominent sample characteristics of the NHANES database make the survey population in this study representative, significantly reducing selection bias. In addition, CHD is a multifactorial disease without any single explicit risk factor. The combination of risk factors selected by different research institutes may yield different results (38). Therefore, this study included as many factors as possible that may affect the occurrence and development of CHD to make the analysis results more accurate and reliable. Nevertheless, it should be acknowledged that this study had some limitations. Firstly, NHANES is a cross-sectional survey. Due to its retrospective nature, further longitudinal study data is needed to prove the causal relationship between CHD and BC. In addition, NHANES uses self-reported questionnaires to collect the history of BC, CHD, hypertension, hypercholesterolemia, and diabetes, which inevitably introduces recall and self-reported bias. Thirdly, the connection between BC and CHD may be a complex composite network relationship. Although we tried to adjust for potential confounding factors, it was inevitable to overlook some factors that may play a role in the pathogenesis of CHD. Finally, the lack of detailed BC information from NHANES also affects the risk of CHD. For example, data on specific treatments for BC (such as surgery, radiotherapy, and chemotherapy). Moreover, BC survivors with different subtypes, grades, and stages may have varying risks of developing CHD, which needs to be further explored in future research.

## 5. Conclusions

This study showed a significant correlation between female BC and an increased risk of CHD. This indicated that clinical doctors should strengthen the long-term management of CHD risk factors for BC survivors and provide early intervention. It may be appropriate to screen specific survivors based on risk factors and inflammatory burden (39). We must work together to develop more effective BC interventions, identify high-risk subtypes of BC, and further investigate the biological and behavioral mechanisms that link BC with CHD.

## Data Availability

Publicly available datasets were analyzed in this study. This data can be found here: https://www.cdc.gov/nchs/nhanes/index.htm.
